# Optimal Energy Scheduling Based on Jaya Algorithm for Integration of Vehicle-to-Home and Energy Storage System with Photovoltaic Generation in Smart Home

**DOI:** 10.3390/s22041306

**Published:** 2022-02-09

**Authors:** Min Wang, Modawy Adam Ali Abdalla

**Affiliations:** 1 College of Energy and Electrical Engineering, Hohai University, Nanjing 211100, China; 2 Electrical Engineering Department, College of Engineering Science, Nyala University, Nyala 63311, Sudan

**Keywords:** smart home, electric vehicle, energy storage system, photovoltaic generation, vehicle-to-home (V2H), Jaya algorithm

## Abstract

With the emerging of the smart grid, it has become easier for consumers to control their consumption. The efficient use of the integration of renewable energy sources with electric vehicle (EV) and energy storage systems (ESSs) in the smart home is a popular choice to reduce electricity costs and improve the stability of the grid. Therefore, this study presents optimal energy management based on the Jaya algorithm for controlling energy flow in the smart home that contains photovoltaic generation (PV), integrated with ESS and EV. The objective of the proposed energy management is to reduce electricity cost while meeting the household load demand and energy requirement for the EV trip distance. By using the Jaya algorithm, the modes of home-to-vehicle (H2V) and vehicle-to-home (V2H) are controlled, in addition to controlling the purchase of energy from the grid and sale of the energy to the grid from surplus PV generation and ESS. Before EV participation in the V2H process, the amount of energy stored in the electric vehicle battery will be verified to be more than the energy amount required for the remaining EV trip to ensure that the required energy for the remaining EV trip is satisfied. Simulation results highlight the performance of the optimal energy scheduling to achieve the reduction of the daily electricity cost and meeting of load demand and EV energy required. The simulation results prove that optimal energy management solutions can be found with significant electricity cost savings. In addition, Jaya is compared with the particle swarm optimization (PSO) algorithm in order to evaluate its performance. Jaya outperforms PSO in terms of achieving optimal energy management objectives.

## 1. Introduction

Nowadays, the increase in energy consumption has become one of the problems that face the power network, especially in the residential sector due to population growth and urbanization, in addition to the use of many appliances by several homes at the same time, which in turn leads to the peaks load [1,2]. Moreover, with the advent of electric vehicles as new technology, carbon emissions have decreased in the transportation sector. In contrast, electricity consumption has increased in the energy sector due to the dependence of electric vehicles on electricity energy as it is the main source [3,4,5]. A recent report indicated that the number of EVs has reached 13 million in 2021 and is expected to exceed 73 million by 2025 [6]. The increase in the number of EVs may increase the peak demand, especially in the residential sector, because electric vehicles are considered among loads of high energy consumption. The peak demand affects the grid flexibility and creates an imbalance between generation and demand. To balance generation and demand, utility companies offer a program called demand response (DR). The demand response program urges the consumers to shift their consumption from peak period to off-peak period (lower electricity price time), depending on the prices provided by utility companies such as time of use (TOU) and real-time pricing (RTP) [7].

On the other hand, the inclusion of renewable energy sources such as solar energy along with the grid contributes to reducing the peak and enhances the flexibility of the grid in addition to the economic benefits due to photovoltaic electricity price subsidy and decrease of PV module cost [8]. However, due to the variability of the available solar energy, the generation period sometimes does not coincide with the period of energy demand. To avoid the intermittent generation of PV, solar energy is used with battery storage [9]. Storage in both stationary battery and electric vehicle batteries has spread because of a reduction in their prices. Although the spread of EV is considered a challenge to the power grid, if it is used in a smart way, such as the technology of the vehicle to anything in conjunction with the stationary battery, it can reduce peak demand and electricity costs.

In contrast, the challenge is how to manage the energy for combining storage in both stationary battery and electric vehicle batteries with grid-connected PV. Therefore, energy management strategies represented by demand-side management (DSM) techniques are significantly important to deal with these challenges. Energy management strategies that rely on the DSM technique operate to match generation with demand by managing the appliances energy consumption on the user side and controlling stationary battery, electric vehicle battery, and PV generation [10]. There are many advantages that DSM techniques provide to grid utilities and consumers, such as reducing peak demand, reducing consumers energy costs, improving the system load curve, and maximizing the use of renewable energy sources. Therefore, this study will focus on optimal scheduling of grid-connected photovoltaic system with stationary battery and EV under a TOU program, in addition to how homeowners schedule their consumption, storage, and sale of surplus energy to the grid so that energy is managed in an optimal way and electricity costs are reduced.

The rest of this study is arranged as follows: The literature review is introduced in Section 2. Section 3 describes system structure and mathematical formulation. Optimal energy scheduling based on the Jaya algorithm is detailed in Section 4. Section 5 discusses the simulation results of the optimal energy scheduling. Lastly, Section 6 elaborates the conclusion.

## 2. Literature Review

The problem of optimal scheduling of consumption, storage, and/or generation in smart homes is considered an optimization problem that is not easy to control. Therefore, several studies have been presented on optimization methods for optimal energy optimal scheduling in smart homes, such as genetic algorithm (GA) [11], mixed-integer linear programming (MILP) [12], mixed-integer nonlinear programming (MINLP) [13], stochastic dynamic programming (SDP) [14], convex programming (CP) [15], shuffled frog leaping algorithm (SFLA) [16], multi-agent system (MAS) [17], and particle swarm optimization (PSO) [18]. In order to determine optimal load energy consumption under a dynamic pricing environment in residential buildings, the genetic algorithm (GA) is proposed with the purpose of reducing the total energy costs [11]. The framework of mixed-integer linear programming (MILP) under the home energy management system (HEMS) model was presented in [12], to limit the peak power with the possibility of bidirectional use of the electric vehicle and energy storage system. Small-scale PV generation, bidirectional energy storage system, V2H, and V2G capabilities, and different demand response strategies were integrated into the proposed HEMS model. In [13], mixed-integer nonlinear programming (MINLP) was formulated for scheduling battery and home appliances under the TOU tariff in the smart home to reduce cost, peak shaving, and valley filling. In [14], the study examined stochastic energy management for the smart home associated with sustainable energy supplies (PV) and the local energy storage opportunity provided by vehicle electrification (EV). Random-variable models such as PV generation and home energy consumption predictive models and Markov Chain model of EV mobility are developed. The stochastic energy management problem was formulated using optimal stochastic dynamic programming (SDP) to manage the power flow between energy sources and reduce the energy cost under TOU while satisfying EV charging requirements and home power demand. In [15], an optimization framework for sizing components and efficient energy use in a residential building with electric vehicle, ESS, and photovoltaic generation was devised. The optimization framework was formulated as a convex programming (CP) problem to efficiently control and optimize the ESS parameters. Vehicle-to-home (V2H) mode, home-to-vehicle (H2V) mode, and buying of electric energy to charge the energy storage system from the grid were controlled based on the convex programming (CP) algorithm.

In [16], the shuffled frog leaping algorithm (SFLA) was designed for optimal scheduling of photovoltaic generation (PV), energy storage system, electric vehicle (EV), and the electric heater (EH) in the smart home through HEMS. The suggested algorithm minimized the daily energy consumption cost and met the electrical and thermal loads. In [17], an efficient real-time embedded system and energy management (RT-ES-EM) was developed in the smart home by using a new multi-agent system (MAS). The proposed algorithm seeks to optimize electricity production without interruption in the hybrid-system-contained solar system with fuel cell backup system (FC). The integration of the electric vehicle (EV) as energy storage systems (ESSs) with uninterruptible power supply (UPS) under demand response help was introduced to reduce electricity bills while maintaining user comfort in the smart home. Electric water heater (EWH), an electric water pump (EWP), heating ventilating, and air conditioning (HVAC) were taken as home appliances which are scheduled by an algorithm of particle swarm optimization (PSO) [18]. In [19], a robust swarm-based optimization algorithm inspired by the grey wolf lifestyle, called grey wolf optimizer (GWO), was adapted to address power scheduling problems in a smart home (PSPSH). PSPSH is formulated as a multi-objective optimization problem to minimize the cost of the power consumed by home appliances, balancing the power consumed during a time horizon, particularly at peak periods, and maximizing the satisfaction level of users. Furthermore, grey wolf optimizer (GWO) and artificial bee colony (ABC) optimization algorithm under ToU pricing scheme based on the Internet of energy have been proposed to define the rates for shoulder-peak and on-peak hours and reduce demand in smart homes [20].

Moreover, several hybrid optimization algorithms have been proposed for optimal energy scheduling in smart homes, for instance, in [21], the HEMS model is designed based on four heuristic algorithms: genetic algorithm (GA), wind driven optimization (WDO), binary particle swarm optimization (BPSO), and bacterial foraging optimization algorithm (BFOA). In addition, a hybrid algorithm called genetic BPSO (GBPSO) is suggested for optimal scheduling of household appliances under real-time pricing (RTP), aiming to reduce the electricity bill and peak-to-average ratio (PAR). In [22], authors proposed a home energy management (HEM) strategy modeled as a mixed-integer linear programming (MILP) with a general algebraic modeling system (GAMS). The proposed strategy reduces electricity cost and compensates reactive power at the grid integration point. The proposed HEM works on scheduling the charging and discharging of the ESS and EV, in addition to scheduling shiftable loads (SL). An algorithm called quadratic binary particle swarm optimization (QBPSO) has been proposed for scheduling household appliances in a smart home equipped with variable renewable energy sources (wind and solar). The proposed algorithm works on optimal scheduling of appliances under both RTP and TOU tariffs to reduce electricity bills with consideration of consumers’ comfort [23]. A hybrid algorithm called firefly lion algorithm (FLA) has been proposed for reducing the cost of energy and the waiting time of consumers or end-users on the university campus [24].

Considering the valuable contributions introduced through the above-proposed optimization algorithms in the HEMS model, most mathematical algorithms, such as MILP and MINLP, have computational complexity or increased system complexity. On the other hand, the heuristic algorithms mentioned above, such as GA and PSO, require algorithmic-specific control parameters that can affect performances and increase computational time. Therefore, optimization algorithms that do not need any algorithmic-specific parameters for their execution and functioning, but just require common parameters such as population size and generation size, have recently been used in solving optimization problems, for example, the Jaya algorithm. Jaya is a simple, powerful, and new optimization algorithm developed by Rao for solving constrained and unconstrained optimization problems (which only requires common control parameters) [25]. Many studies have been proposed for optimal energy scheduling in smart homes by using the Jaya algorithm in the HEMS model, as in [26,27]. In [26], the Jaya algorithm was proposed for optimal energy scheduling in smart homes integrated with the photovoltaic system (PV) and energy storage system (ESS). The proposed algorithm operates on optimal scheduling of ESS and home appliances in response to critical peak price (CPP) and time-of-use (TOU) to reduce the cost of electricity. A multi-objective self-adaptive multi-population algorithm has been suggested for optimal energy scheduling in smart buildings equipped with rooftop PV and ESS. The proposed algorithm based on the Jaya algorithm was developed to minimize carbon emission and electricity price [27]. Table 1 shows a summary of techniques, objectives, features, and limitations of the literature.

To overcome the shortcomings of the previous studies, this study proposes a Jaya framework for the rapidly and efficiently optimal energy scheduling of a smart home with the ESS, EV, and photovoltaic generation. The aim of this work is to reduce the daily cost of electricity and meet the household demand and energy requirement for the EV trip distance. The major contributions of this study are summarized as follows:

- Indeed, the main contribution of this work was to monitor the energy flow of the studied home by using the Jaya algorithm according to the following constraints: TOU electricity price signal, the specific daily load profile, PV generation profile, EV constraints, ESS constraints, and power balance, exchange, and flow constraints.
- One of the advantages that distinguish this work is that it takes into account the effect of EV travel distance when using the EV in vehicle-to-home (V2H) mode. Therefore, before EV participation in the V2H process, the amount of energy stored in the electric vehicle battery will verify to be enough for the remaining vehicle trip distance to ensure that the required energy for the electric vehicle trip distance is satisfied based on vehicle trip distance. The verification of required energy for the electric vehicle trip distance has not been performed in any literature.
- The proposed model elaborates that by using the Jaya algorithm, the modes of home-to-vehicle (H2V) and vehicle-to-home (V2H) are controlled, in addition, to controlling the purchase of energy from the grid and sale of the energy to the grid from PV generation and ESS.

## 3. System Structure and Mathematical Modeling

### 3.1. Studied System Structure

**Figure 1:**
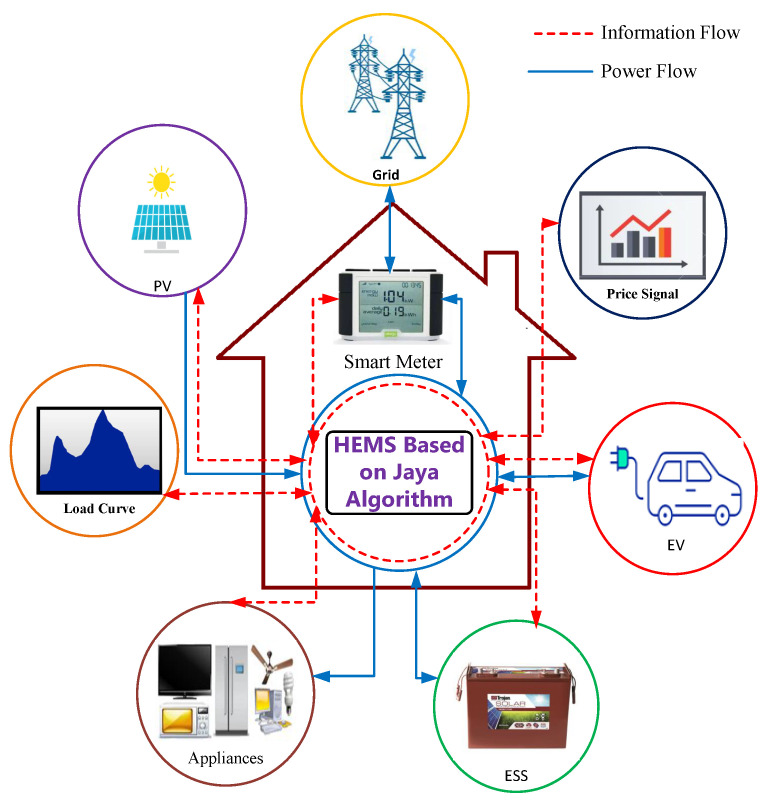
Configuration of smart home.

**Figure 2:**
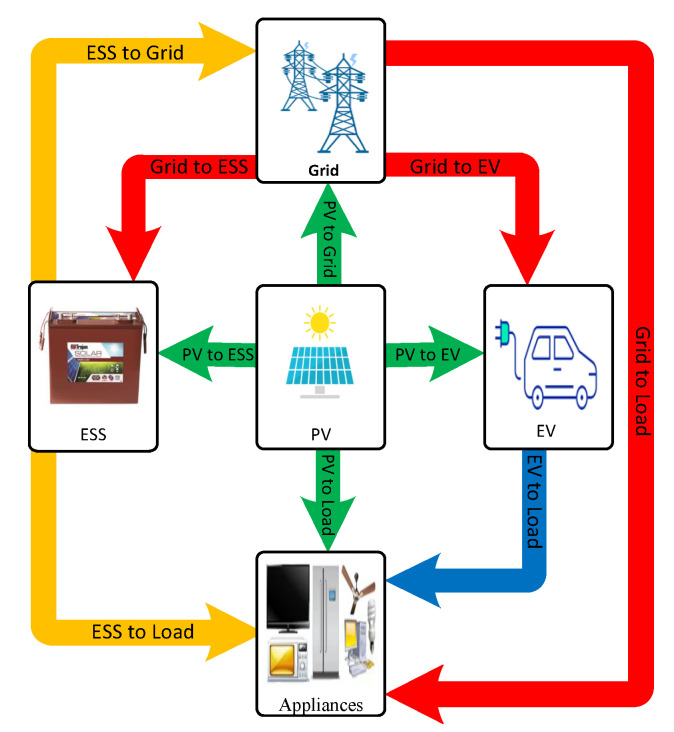
Energy exchange paths between components of the smart home.

The configuration of the typical smart home is depicted in Figure 1. Besides the utility grid, the smart home under study includes a load profile, electricity price signal, solar radiation, photovoltaic system (PV), an electric vehicle (EV), household appliances, and an energy storage system. In order to achieve an optimal energy scheduling between the power grid and the studied smart home, a smart meter is required to collect information about the power quantity required or supplied to the utility grid [28]. In addition, the smart home energy management system (HEMS) is in charge of optimal energy exchange between smart house components and the utility grid [29,30]. The optimal control problem of energy scheduling between the power grid and the studied smart home entails control inputs as TOU electricity price signal, the specific daily load profile, PV generation profile, EV parameters, and ESS parameters. The control decisions are the power flows between the grid and the studied smart home, state of charge (SOC) of EV and ESS, which have battery charging and discharging power as control variables, and the arrival and departure time of the electric vehicle. The main objective is to reduce the daily cost of electricity and meet the household demand and energy requirement for the EV trip distance, taking into account the necessary constraints. This problem is of great importance because worldwide research on DSM, of which the main purpose is to match generation with demand by managing and controlling the energy consumption, energy storage, and generation on the user side, has opened new possibilities for advanced planning and control of consumption storage and generation, especially at the residential level in which the scheduling of energy consumption storage and generation can play a major role. Figure 2 displays the different combinations of energy exchange paths between components of the smart home.

### 3.2. Mathematical Modeling

#### 3.2.1. Objective Function

The objective function of this study is the economic optimization of the smart home performance, aiming to minimize the total cost of electricity. The objective function is composed of three parts. The first part is the cost of power bought from the grid. The second part is the daily cost of PV and ESS installation and the daily EV battery degradation cost. The third part is the revenue of power sold to the grid.

(1) $$MinHcost=Cost_{P}^{buy}+Cost_{PV,ESS,EV}^{installation}-revenue_{P}^{sell}$$

(2) $$Cost_{P}^{buy}=\sum_{t=1}^{24}\lambda_{t}\left[P_{GL,t}-P_{PVL,t}-P_{ESSL,t}-P_{EVL,t}\right]+\sum_{t=1}^{24}\lambda_{off}\left[P_{GESS,t}+P_{GEV,t}\right]$$

(3) $$Cost_{PV,ESS,EV}^{installation}=Cost_{PV}^{\mathrm{capital}}+Cost_{ESS}^{\mathrm{capital}}+Cost_{EV}^{degradation}$$

(4) $$revenueP_{grid}^{sell}=\sum_{t=1}^{24}\gamma \left[\lambda_{t}P_{PVG,t}+\lambda_{k}P_{ESSG,t}\right]$$

where $P_{GL,t}$ is power from the grid-to-load (kW), $P_{GESS,t}$ represents the power from the grid-to-ESS (kW), $P_{GEV,t}$ represents power from the grid-to-EV (kW), $P_{PVG,t}$ represents the power from the PV-to-grid (kW), $P_{ESSG,t}$ represents the power from the ESS-to-grid (kW), $P_{PVL,t}$ represents the power from the PV-to-load (kW), $P_{ESSL,t}$ represents the power from the ESS-to-load (kW), $P_{EVL,t}$ represents the power from the EV-to-load (kW), and gamma is the contracted ratio for selling power to the grid.

The TOU pricing signal $\lambda_{t}$ is used in the economic optimization of the smart home performance, peak price $\lambda_{k}$, mid-peak price $\lambda_{m}$, and off-peak price $\lambda_{off}$. The daily electricity price during different periods can be formulated as follows.

(5) $$\lambda_{t}=\left\{\begin{array}{c} \begin{array}{cc} \lambda_{k}, & \forall t=t_{k} \end{array}\\ \begin{array}{cc} \lambda_{mid}, & \forall t=t_{mid} \end{array}\\ \begin{array}{cc} \lambda_{off}, & \forall t=t_{off} \end{array} \end{array}\right.$$

The $Cost_{PV}^{\mathrm{capital}}$ and $Cost_{ESS}^{\mathrm{capital}}$ represent the daily capital cost of the PV installation and the daily capital cost of ESS installation, which are formulated as in Equations (6) and (7) respectively [31], and $Cost_{EV}^{degradation}$ represents the EV battery degradation cost due to V2H, which is formulated as in Equation (8) [32].

(6) $$Cost_{PV}^{\mathrm{capital}}=C_{PV}\frac{i{\left(1+i\right)}^{N}}{{\left(1+i\right)}^{N}-1}\frac{Z_{PV}}{N_{days}}$$

(7) $$Cost_{ESS}^{\mathrm{capital}}=C_{ESS}\frac{i{\left(1+i\right)}^{N}}{{\left(1+i\right)}^{N}-1}\frac{Z_{ESS}}{N_{days}}$$

(8) $$Cost_{EV}^{degradation}=\frac{C_{EV}}{C_{f}DOD}\left(Z_{EV}DOD-E_{EV}^{R}\right)\eta_{EV}^{d}$$

where $C_{PV}$ is one-time installation cost of PV ($/kW), *i* is the interest rate, *N* is lifetime, $N_{days}$ is the total number of days during the year, $Z_{PV}$ is rated capacity of PV array size (kW), $C_{ESS}$ is the one-time installation cost of ESS ($/kWh), $Z_{ESS}$ is rated capacity of ESS (kWh), $C_{EV}$ is the replacement cost of EV battery, $C_{f}$ is the full cycles number during battery lifespan, DOD is the total number depth of discharge, $Z_{EV}$ is rated capacity of EV battery (kWh), $E_{EV}^{R}$ is the required energy for the EV travel distance in the typical day (kWh), and $\eta_{EV}^{d}$ is the EV battery discharge efficiency.

The required energy for the electric vehicle trip distance can be represented by the following equation [33].

(9) $$E_{EV}^{R}=\eta_{V}D$$

where $\eta_{V}$ represents vehicle efficiency (kWh/km), and *D* represents the vehicle travel distance (km).

#### 3.2.2. Operational Constraints of PV

The PV output constraint depends on power out from the PV array. The PV array consists of many solar cells that are interconnected in series and/or parallel to convert sunlight into DC power by a photovoltaic effect. The hourly power output from the PV generator of the specific area size can be formulated as follows:

(10) $$P_{PV}=\eta_{PV}Z_{PV}I_{PV}$$

where $\eta_{PV}$ represents PV generator efficiency and $I_{PV}$ represents the solar radiation (kWh/m ${}^{2}$).

The efficiency of the PV generator is given by [34].

(11) $$\eta_{PV}=\eta_{R}\left[1-0.9\rho \left(\frac{I_{PV}}{I_{PV,NT}}\right)\left(T_{C,NT}-T_{A,NT}\right)-\rho \left(T_{A}-T_{R}\right)\right]$$

where $\eta_{R}$ is the PV generator efficiency measured at reference cell temperature $T_{R}$, i.e., under standard test conditions (25 ^∘^C); ρ is the temperature coefficient for cell efficiency (typically 0.004–0.005/^∘^C); $I_{PV,NT}$ is the average hourly solar irradiation incident on the array at NT (0.8 kWh/m^2^); $T_{C,NT}$ (typically 45 ^∘^C) and $T_{A,NT}$ (20 ^∘^C) are, respectively, the cell and ambient temperatures at NT test conditions.

The constraint of PV output in this study that lies in PV power generation should be greater than or equal to the amount of PV power for immediate customer use, the amount of PV power used to charge ESS and EV, and the amount of PV power sold to the grid.

(12) $$P_{PVL,t}+P_{PVESS,t}+P_{PVEV,t}+P_{PVG,t}\leq P_{PV,t}$$

#### 3.2.3. Operational Constraints of ESS

The state of charge of ESS constraint: Due to the limited capacity of the ESS, the ESS state of charge at time interval t will change depending on the residual energy from the past period and the charging and discharging in that interval. According to the state of charge of ESS at the past period, the state of charge of the current period can be formulated as

(13) $$SOC_{ESS,t}=SOC_{ESS,t-1}+\frac{1}{Z_{ESS}}\left[\eta_{ESS}^{c}P_{ESS,t}^{c}-\frac{P_{ESS,t}^{d}}{\eta_{ESS}^{d}}\right]$$

where $SOC_{ESS,t}$ is the ESS SOC at time t, $SOC_{ESS,t-1}$ is the SOC of ESS in past period, $P_{ESS,t}^{c}$ represents charging power of ESS (kW), $P_{ESS,t}^{d}$ represents discharging power of ESS (kW), $Z_{ESS}$ represents the ESS rated capacity (kWh), and $\eta_{ESS}^{c}$ and $\eta_{ESS}^{d}$ represent charging and discharging efficiency of ESS, respectively.

ESS SOC boundary constraint: To maintain the state of charge of the ESS within the minimal and maximal allowable capacity, the state of charge of ESS is constrained as follows.

(14) $$SOC_{ESS,\min}\leq SOC_{ESS,t}\leq SOC_{ESS,\max}$$

where $SOC_{ESS,\max}$ is the maximum SOC of ESS, and $SOC_{ESS,\min}$ is the minimum SOC of ESS.

The constraint of sale of energy stored in the ESS to the grid: The energy stored in the ESS must be sold to the grid at peak price periods only.

(15) $$P_{ESSG,t}=\left\{\begin{array}{c} \begin{array}{cc} 0\leq P_{ESSG,t}\leq P_{{}_{ESSG}}^{\max}, & \forall \lambda_{t}=\lambda_{k}\\ 0, & otherwise \end{array} \end{array}\right.$$

The constraint of charging ESS from the grid: The ESS must be charging from the grid at off-peak price periods only.

(16) $$P_{GESS,t}=\left\{\begin{array}{c} \begin{array}{cc} 0\leq P_{GESS,t}\leq P_{{}_{GESS}}^{\max}, & \forall \lambda_{t}=\lambda_{off}\\ 0, & otherwise \end{array} \end{array}\right.$$

#### 3.2.4. Operational Constraints of EV

Similar to ESS, the EV battery state of charge will change due to charging and discharging depending on the remaining energy from the previous period. The following equation expresses the state of charge of EV battery in the current period depending on the state of charge of EV battery at the past period.

(17) $$SOC_{EV,t}=SOC_{EV,t-1}+\frac{1}{Z_{EV}}\left[\eta_{EV}^{c}P_{EV,t}^{c}-\frac{P_{EV,t}^{d}}{\eta_{EV}^{d}}\right]$$

where $SOC_{EV,t}$ represents EV battery SOC at time t, $SOC_{EV,t-1}$ represents state of charge of EV battery at the past period, $P_{EV,t}^{c}$ represents charge power of EV (kW), $P_{EV,t}^{d}$ represents discharge power of EV (kW), $Z_{EV}$ represents the EV battery rated capacity (kWh), and $\eta_{EV}^{c}$ and $\eta_{EV}^{d}$ represent charging and discharging efficiency of EV battery, respectively.

In addition, similar to the ESS SOC constraint, the SOC of the EV should be within a certain range represented by the maximum and minimum state of charge levels as follows.

(18) $$SOC_{EV,\min}\leq SOC_{EV,t}\leq SOC_{EV,\max}$$

where $SOC_{EV,\max}$ is the maximum SOC of EV battery, and $SOC_{EV,\min}$ is the minimum SOC of EV battery.

The constraint of charging EV from the grid: The EV must only be charging from the grid at off-peak price periods.

(19) $$P_{GEV,t}=\left\{\begin{array}{c} \begin{array}{cc} 0\leq P_{GEV,t}\leq P_{{}_{GEV}}^{\max}, & \forall \lambda_{t}=\lambda_{off}\\ 0, & otherwise \end{array} \end{array}\right.$$

The constraint of providing the required energy for the electric vehicle trip distance in case of EV battery discharging: Before the participation of EV in the V2H process, the amount of energy stored in the electric vehicle battery will be verified to be enough for the remaining vehicle trip distance to ensure that the required energy for the electric vehicle trip distance is satisfied based on vehicle trip distance. Therefore, the state of charge of EV battery at departure time must be equal or higher than SOC required for the EV trip distance.

(20) $$\begin{array}{c} \begin{array}{cc} SOC_{EV,t}\geq SOC_{EV,R1}, & \forall t=t_{d1} \end{array} \end{array}.$$

(21) $$\begin{array}{c} \begin{array}{cc} SOC_{EV,t}\geq SOC_{EV,R2}, & \forall t=t_{d2} \end{array} \end{array}.$$

where $SOC_{EV,R1}$ is the SOC required for the EV trip distance at the first departure time $t_{d1}$, and $SOC_{EV,R2}$ is the SOC required for the remaining EV trip distance at the second departure time $t_{d2}$.

#### 3.2.5. Power Balance Constraint

The power of grid, PV, ESS, and EV must supply household power demand as follows.

(22) $$P_{GL,t}+P_{PVL,t}+P_{ESSL,t}+P_{EVL,t}=P_{L,t}$$

From Equation (22), the bought power from the grid and power generated by PV besides the discharged power from ESS and EV battery should be equal to the home power demand, where $P_{L,t}$ is the household power demand at time t (kW).

#### 3.2.6. Power Exchange Constraints

Electricity energy should not be sold to the grid and bought from the grid at the same time, which can be formulated and satisfied using a binary variable (β). From Equations (23) and (24), it is worth noting that the home can draw power from the grid when β is equal to 1 and sell back power to the grid when β is equal to 0.

(23) $$P_{GL,t}+P_{GESS,t}+P_{GEV,t}\leq \beta P_{\max}^{grid}$$

(24) $$P_{PVG,t}+P_{ESSG,t}\leq \left[1-\beta \right]P_{\max}^{sell}$$

where $P_{\max}^{grid}$ is the maximum power that can be drawn from the grid (kW), and $P_{\max}^{sell}$ is the maximum power that can be sold back to the grid (kW).

#### 3.2.7. Power Flow Constraints

For safety and other physical reasons, power flow from each source must be non-negative and less than the maximum allowable value as

(25) $$0\leq P_{GL,t}\leq P_{GL}^{\max}$$

(26) $$0\leq P_{GEV,t}\leq P_{GEV}^{\max}$$

(27) $$0\leq P_{GESS,t}\leq P_{GESS}^{\max}$$

(28) $$0\leq P_{PVL,t}\leq P_{PVL}^{\max}$$

(29) $$0\leq P_{PVEV,t}\leq P_{PVEV}^{\max}$$

(30) $$0\leq P_{PVESS,t}\leq P_{PVESS}^{\max}$$

(31) $$0\leq P_{PVG,t}\leq P_{PVG}^{\max}$$

(32) $$0\leq P_{ESSL,t}\leq P_{ESSL}^{\max}$$

(33) $$0\leq P_{ESSG,t}\leq P_{ESSG}^{\max}$$

(34) $$0\leq P_{EVL,t}\leq P_{EVL}^{\max}$$

## 4. Optimal Energy Scheduling Based on Jaya Algorithm

In this work, the problem introduced in Section 3.1 is the optimal control problem of energy scheduling between the power grid and the studied smart home, which belongs to scheduling problems. Most traditional mathematical algorithms, such as MILP and MINLP, are not adequate to control the scheduling optimization, and have computational complexity or increase system complexity. Therefore, metaheuristic algorithms such as GA, PSO, and Jaya are more and more used in scheduling optimization. In most cases, they give a good solution in a reasonable time for larger problems. However, some of the metaheuristic algorithms, such as GA and PSO, require algorithmic-specific control parameters that can affect performances and increase computational time. Therefore, to address this, we apply a metaheuristic Jaya algorithm to actualize our set of objectives, which do not need any algorithmic-specific parameters for their execution and functioning but just require common parameters such as population size and generation size. The Jaya algorithm is proposed and the result is compared with the PSO algorithm.

The theoretical and algorithmic aspects of Jaya are detailed in [25]. Suppose there is F(x) as an objective function that can be maximized or minimized. In any iteration t, let j represent the variables number (m = 1, 2, *…*, j) and k represent population size or candidate number (n = 1, 2, *…*, k). In addition, assume that the best value of the objective function (F(x)best) is found from the best candidate among all candidate solutions and the worst value of the objective function (F(x)worst) is found from the worst candidate among all candidate solutions. Let X_m,n,t_ be the value of mth variable for nth candidate at the tth iteration, then the value will be modified by the following equation:

(35) $$X_{m,n,t}^{{}^{\prime }}=X_{m,n,t}+r_{1}\left(X_{m,best,t}-\left|X_{m,n,t}\right|\right)-r_{2}\left(X_{m,worst,t}-\left|X_{m,n,t}\right|\right)$$

where $X_{m,best,t}$ represents the variable m best value obtained for the best candidate, $X_{m,worst,t}$ is the variable m worst value obtained for the worst candidate, $X_{m,n,t}^{{}^{\prime }}$ the modified value of $X_{m,n,t}$, $r_{1}$ and $r_{2}$ are random values between zero and one. The $r_{1}\left(X_{m,best,t}-\left|X_{m,n,t}\right|\right)$ is the term of moving near to the best solution and $r_{2}\left(X_{m,worst,t}-\left|X_{m,n,t}\right|\right)$ is term of avoiding the worst solution. After that, the new solution value, found by $X_{m,n,t}^{{}^{\prime }}$, and previous solution value found by $X_{m,n,t}$ will be compared, and if the new solution value is better than the previous solution value, the new value will be accepted and will replace the previous solution. Otherwise, the previous solution will be kept and it will continue to work in that manner until the end of iteration t. The pseudocode of Jaya is outlined in Algorithm 1, which is simple to understand.
**Algorithm 1:** Pseudocode of Jaya.1:**Input:** Input: objective function (f), Population size (n)Number of design variables (m), Lower and Upper bounds (Lb,Ub),and Maximum number of iterations (Maxiter)2:**Output:** best solution and best objective function value3:Initialize the population within lower and upper bounds randomly4:Evaluation of fitness values5:iter = 16:**while** iter < Maxiter7:Find the best and worst solution among the current population8:       **for** n = 1 to k do9:            **for** m = 1 to j do10:               Update the solution according to Equation (35)11:              **if** $X_{m,n,t}^{{}^{\prime }}$ < Lb12:                  $X_{m,n,t}^{{}^{\prime }}$ = Lb13:              **else if** $X_{m,n,t}^{{}^{\prime }}$ > Ub14:                  $X_{m,n,t}^{{}^{\prime }}$ = Ub15:              **else**16:                   $X_{m,n,t}^{{}^{\prime }}$ = $X_{m,n,t}^{{}^{\prime }}$17:              **end if**18:           **end for**19:           **if** solution f($X_{m,n,t}^{{}^{\prime }}$) better than f($X_{m,n,t}$)20:              $X_{m,n,t}$ =$X_{m,n,t}^{{}^{\prime }}$ (replace old solution by new solution)21:           **else**22:              $X_{m,n,t}$ =$X_{m,n,t}$ (keep old solution)23:           **end if**24:       **end for**25:iter = iter + 126:**end while**

**Figure 3:**
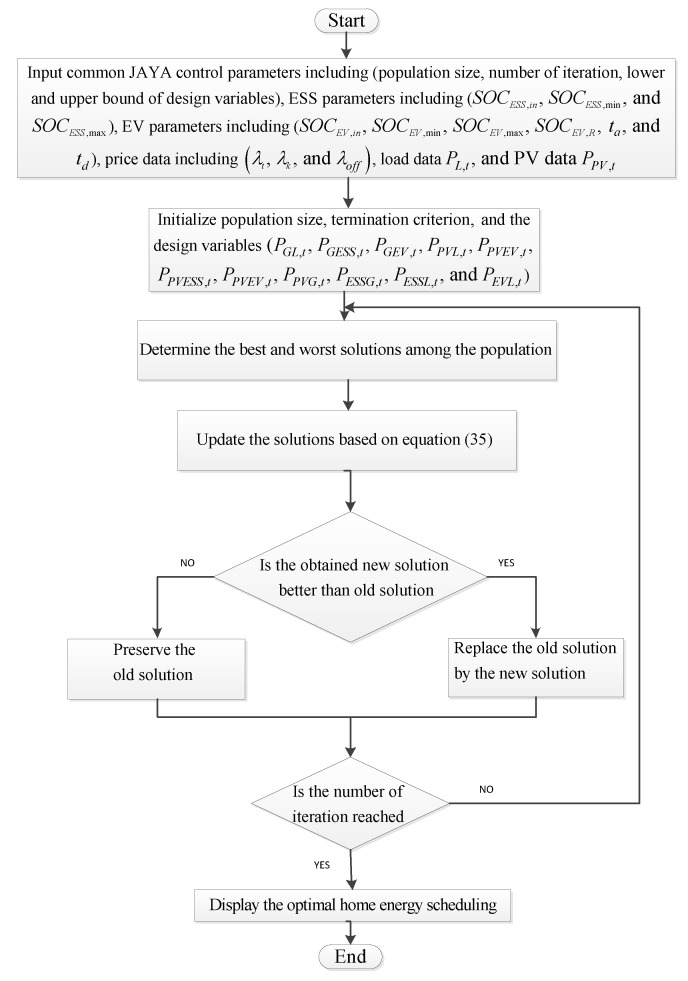
The flowchart of optimal energy scheduling based on Jaya algorithm.

The objective function of the studies system in Equation (1) is used as Jaya objective function, which is of great interest to the household economic optimization performance, which is formulated to minimize the total cost of electricity. The constraints in this study are represented by PV constraints (Equation (12)), the ESS constraints (Equations (13)–(16)), the EV battery constraints (Equations (17)–(21)), the power balance constraints (Equation (22)), power exchange constraints (Equations (23) and (24)), and power flow constraints (Equations (25)–(34)). The number of design variables in this work is equal to the number of power flow equations, which include $P_{GL,t}$, $P_{GESS,t}$, $P_{GEV,t}$, $P_{PVL,t}$, $P_{PVEV,t}$, $P_{PVESS,t}$, $P_{PVG,t}$, $P_{ESSG,t}$, $P_{ESSL,t}$, and $P_{EVL,t}$. The flowchart in Figure 3 displays the optimal energy scheduling based on the Jaya algorithm for integrating vehicle-to-home and energy storage systems with photovoltaic generation in smart homes.

## 5. Simulation Results and Discussion

This section provides simulation results to verify the proposed algorithm’s performance and efficiency in minimizing the smart home’s daily electricity cost. The computational time of this study is executed within 24 h.

**Figure 4:**
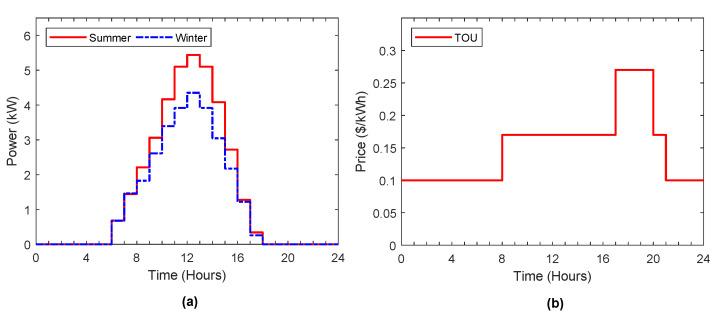
(**a**) Power output curve of solar in summer and winter; (**b**) TOU price signal.

The solar irradiation and temperature data were taken from [35], which provides the solar meteorological data from 1981 to the date of access on 1 November 2021. To simplify, the hourly solar meteorological data during the last five years were utilized to evaluate the average power output curve of solar in summer and winter, which can be depicted in Figure 4a.

The electricity pricing signal for residential customers is shown in Figure 4b, which represents the residential time-of-use rates that refer to the Southern California Edison (SCE) TOU plans [36]. This figure displays the price profile of electricity with TOU in different intervals, which displays off-peak, mid-peak, and peak electricity prices during the time of day. It motivates the customers to charge their ESS and EV at low prices of electricity which can be used later on to feed load demand or sell it back to the grid at high prices of electricity to achieve economic benefits.

**Figure 5:**
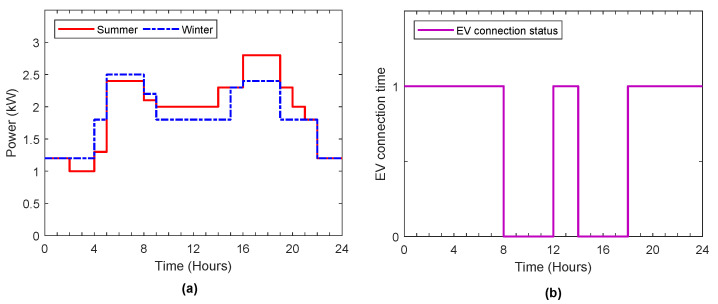
(**a**) Hourly home energy consumption; (**b**) EV connection time.

Near-real-time hourly electricity consumption data was collected from 48 states by the US energy information administration (EIA) [37]. They found that household electricity consumption is highest in the summer months. In contrast, hourly electricity load is less variable during the winter months but peaks in both the morning and the evening. Therefore, in this study, the home energy consumption in summer and winter are predicted as shown in Figure 5a.

PV systems prices in 2021 decreased by 5% compared to the previous year for systems from 2.5 kW to 10 kW. The PV data used in this study are presented in Table 2 [38].

It is supposed that the electric vehicle charges only at home, and the adequate level to charge the EV in the house ranges between 1.5–3 kW [39]. The electric vehicle daily travel distance is considered 40 miles, which is the average travel distance in the United States [40]. Furthermore, it is assumed that the electric vehicle is away from the house at 8:00 a.m., and back at the house at 12:00 p.m., away from the house again at 02:00 p.m., and back at house at 05:00 p.m., as in Figure 5b. Technical and economic data of the EV utilized in this study are taken from [41], and manufacturer specification and economic data of the chosen batteries used in the hybrid system are taken from [42]. Table 3 lists the parameters of EV and ESS used in this simulation. In this work, the interest rate is considered to be 6%. All Jaya parameters used in this simulation are shown in Table 4.

**Figure 6:**
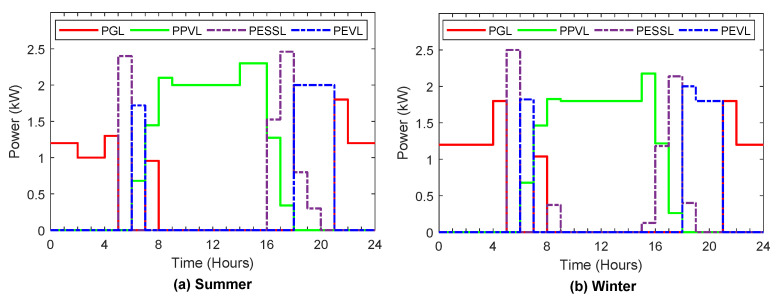
Optimal power scheduling to feed the load demand in summer and winter.

**Figure 7:**
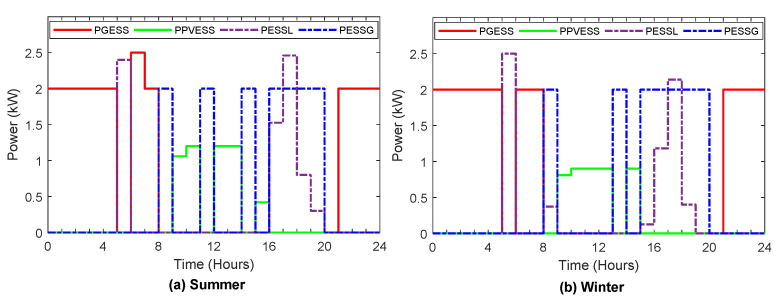
Optimal power flows from/to energy storage system during summer and winter.

**Figure 8:**
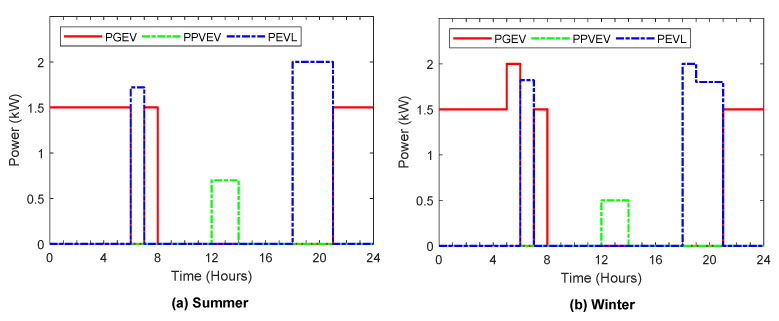
Optimal power flows from/to electric vehicle during summer and winter.

**Figure 9:**
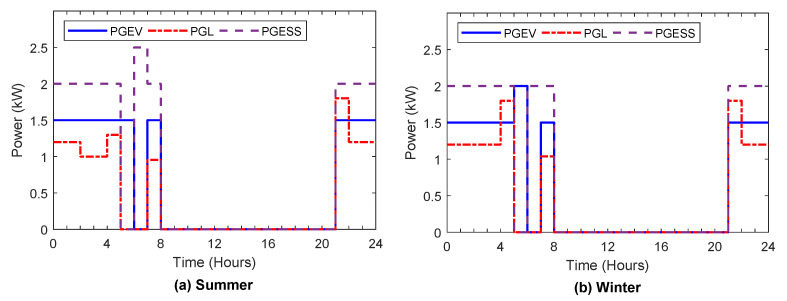
Procured power from grid.

**Figure 10:**
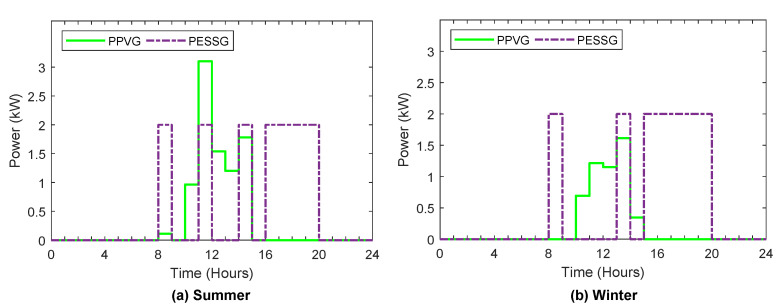
Sold power to the grid.

Figure 6, Figure 7, Figure 8, Figure 9 and Figure 10 display the optimal power flows achieved with the proposed optimal energy scheduling based on the Jaya algorithm.

Figure 6a,b show the optimal power flows to feed the load demand of the customer during the typical day in summer and winter, respectively, which include power from the grid to load, power from PV to demand, power from ESS to demand, and power from EV to demand. During the peak time (high electricity price), the load is fed only by PV generation, ESS, and EV, while the load is fed from the grid during low electricity price or off-peak period only, unless the PV generation is not sufficient to supply the load, and maximum allowable value for feeding the load from ESS or EV is less than the load, or if EV is not available at home, the load will be fed from the grid at peak time.

Figure 7 displays the optimal power flows from/to the energy storage system during the typical day in winter and summer, which include power from ESS to load and grid and power from PV generation and grid to ESS. The ESS is charged from the grid during off-peak intervals. When the PV generation is sufficient and more than the household load, priority is given to charge the ESS from the PV generation in those periods. The energy stored in the ESS is utilized to minimize the daily cost of electricity and satisfy the demand by discharging the ESS to feed the load during peak periods, and periods when PV generation is insufficient, in addition to selling the energy stored in the ESS to the grid when the electricity prices are high.

The optimal power flows from/to EV battery during the typical day in winter and summer are plotted in Figure 8. The electric vehicle power flows include charging EV from the grid during the off-peak periods, charging EV from solar when the solar generation is higher than the load demand, and EV discharging to feed the load. In addition, this involves meeting the load demand through discharging of EV to feed the load demand when the load demand is greater than the generated PV and has not been fed by the ESS, after making sure that the energy stored in the EV battery is more than the amount of energy required for the remaining electric vehicle trip.

Figure 9a,b illustrate procured power from the grid to feed the load demand, ESS, and EV in summer and winter, respectively. According to Figure 9a,b procured power from the grid was reduced during the high electricity price intervals and increased during low electricity prices, which minimized the household’s total cost.

Figure 10a,b illustrate sold power to the grid in summer and winter, respectively, including the power sold to the grid from PV and ESS. It is noted that PV power is sold to the grid as soon as the PV generation is redundant, while the energy stored in the ESS is not sold to the grid unless the electricity prices are high, which increases the income of selling electricity.

**Figure 11:**
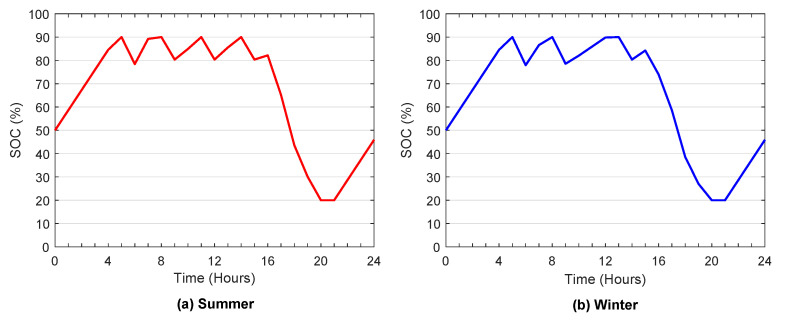
Energy storage system state of charge.

The ESS state-of-charge curve in summer and winter is illustrated in Figure 11a,b, respectively. These figures show that the ESS is in charge and discharge status, and it is noted that the state of charge is increased in the period of high irradiation and off-peak time and decreased at the peak period. In addition, the figures confirm that the SOC is within the maximum and minimum levels during ESS charge and discharge, and the SOC boundary constraints have been satisfied.

**Figure 12:**
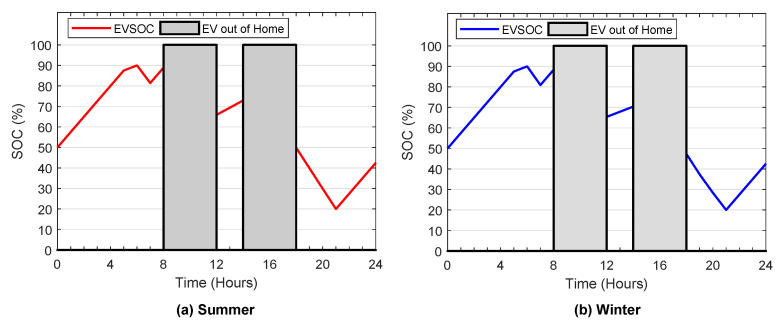
EV state of charge.

Figure 12a,b show EV battery SOC profiles in summer and winter, respectively, and the increase and decrease of EV battery SOC values confirm the charge and discharge cases. From these figures, it is worth noting that the EV battery SOC boundary constraints have been satisfied.

In order to evaluate the performance of the Jaya algorithm and prove its reliability, PSO is adapted and evaluated in terms of achieving optimal energy scheduling objectives. The results obtained by Jaya and PSO are compared to show and evaluate the performance of the algorithms. PSO is examined using the same parameters and constraints used in the studied smart home. Table 5 shows the parameter settings of the PSO.

The results of Jaya and PSO are compared in summer and winter. Table 6 shows the results obtained by Jaya and PSO in summer and winter. The table clearly shows the powerful performance of Jaya in achieving the optimal energy scheduling objectives.

In the summer, without the proposed energy scheduling system, the daily home electricity cost due to the feed of household appliances and EV from the grid is USD 9.27, while after the use of proposed optimal energy scheduling based on the Jaya and PSO algorithms, daily home electricity cost dropped to USD 2.79 and USD 3, which represent 30% and 32% of total energy consumption cost during the summer, respectively (which means that the total energy consumption cost during the summer was reduced by 70% and 68%). On the other hand, in the winter, without the proposed energy scheduling system, the daily home electricity cost due to the feed of household appliances and EV from the grid is USD 8.75. However, after utilizing the Jaya and PSO algorithms with the proposed hybrid system, the daily home electricity cost was reduced to USD 4.0 and USD 4.3, which represent 46% and 49% of total energy consumption cost during the winter, respectively (which means that the total energy consumption cost during the winter was reduced by 54% and 51%).

## 6. Conclusions

This paper introduced optimal energy management based on the Jaya algorithm for the smart home that integrates PV generation of EV and ESS. The aim of the proposed work was to reduce the daily cost of electricity and meet the household demand and energy requirement for the EV trip distance according to the set of constraints: TOU electricity price signal, the specific daily load profile, PV generation profile, EV constraints, ESS constraints, and power balance, exchange, and flow constraints. The Jaya algorithm was used to control modes of home-to-vehicle (H2V) and vehicle-to-home (V2H), in addition to control of the energy purchase from the grid and sale of the energy to the grid from surplus PV generation and ESS. Simulation results highlight the performance.

The simulation results display that, by utilizing optimal energy scheduling based on the Jaya algorithm in a smart home containing electric vehicle, ESS, and photovoltaic generation, the use of PV generation, EV, and ESS was maximized. In addition, it can be noted that ESS and EV play a great role in storing power from the grid during cheap electricity price periods and feeding the load demand and selling it back to the grid during high electricity price periods. Therefore, with the optimal scheduling based on the Jaya algorithm of PV generation, EV, and ESS, the homeowner consumes a minimum grid power amount and minimizes their daily electricity cost.

## Figures and Tables

**Table 1:** Summary of literature.

Techniques	Objectives	DER	DSMR	Limitations
[11] GA	Minimizing overall electricity cost	-	-	Computational complexity and inconsideration of DER and DSMR
[12] MILP	Peak power limitation and cost reduction	PV	ESS and EV	Computational complexity
[13] MINLP	Achieving of cost saving, peak shaving, and valley filling	-	ESS	Ignoring of DER
[14] SDP	Minimize consumer charges and satisfying load and EV energy requirements	PV	EV	System complexity is increased
[15] CP	Home economy maximization and satisfying load and EV energy requirements	PV	ESS and EV	System complexity is increased
[16] SFLA	Minimization of electricity bill and air pollution of the home and balances electricity and natural gas consumption during seasons	PV	ESS and EV	System complexity is increased
[17] RT-ES-EM with MAS	Improvement of electricity production without interruption to provide comfortable services for users	PV	ESS and FC	Implementing of V2H and cost reduction were not considered
[18] PSO	Bill reduction and satisfying user requirement	-	EV as ESS	Ignoring of DER
[19] GWO	Minimize the cost, balance the power consumption, and maximize the satisfaction level of users	-	ESS	Ignoring of DER and EV
[20] GWO and ABC	Reduce the demand	-	-	Inconsideration of DER and DSMR
[21] GA, WDO, BPSO, BFOA, and GBPSO	Reduction of electricity cost and peak- to-average ratio (PAR)	-	-	Ignoring of DER and DSMR
[22] MILP with GAMS	Reduces electricity cost and compensates the reactive power at the grid integration point	PV	ESS and EV	Computational complexity
[23] QBPSO	Electricity bill minimization and maximizes the consumer comfort	Wind and PV	-	Inconsideration of DSMR
[24] FLA	Cost reduction	-	-	Ignoring of DER and DSMR
[26] Jaya	Reduction of electricity cost	PV	ESS	Inconsideration of V2H
[27] PMO- SAMP Jaya	Minimization of economic cost and CO^2^ emissions	PV	ESS	Use of EV as electrical load and ignores the use of V2H

**Table 2:** PV parameters.

Parameter	Value
Lifetime	25 year
PV rated power	6 kW
One-time investment cost	3780 $/kW
Module efficiency	18%

**Table 3:** Parameters associated with the ESS and EV.

Parameters	EV	ESS
Battery capacity	19 kWh	19.68 kWh
Cost	324 $/kWh	250 $/kWh
$SOC_{\max}$	90%	90%
$SOC_{\min}$	20%	20%
Initial SOC	50%	50%
Depth of discharge DOD	80%	80%
Charging efficiency	0.95	0.85
Discharging efficiency	0.95	0.95
Lifetime	2000 cycles	10 years
Vehicle depart time	08:00, 02:00	-
Vehicle arrive time	12:00, 17:00	-
Vehicle efficiency	14 kWh/100 km	-

**Table 4:** Jaya parameters.

Parameter	Value
Population	100
Max iteration	100
Upper bound	1
Lower bound	0

**Table 5:** PSO parameters.

Parameter	Value
Population	100
Max iteration	100
Upper bound	1
Lower bound	0
$W_{max}$	0.9
$W_{min}$	0.4
$C_{1}$	2
$C_{2}$	2

**Table 6:** Comparison between base case, Jaya, and PSO in summer and winter.

Seasons	Cases	Total Daily Cost (USD)	Daily Cost Reduction (%)
	Without the optimal energy scheduling	9.27	Base case
Summer	Optimal energy scheduling based on Jaya	2.79	70
	Optimal energy scheduling based on PSO	3.0	68
	Without the optimal energy scheduling	8.75	Base case
Winter	Optimal energy scheduling based on Jaya	4.0	54
	Optimal energy scheduling based on PSO	4.3	51

## Data Availability

Not applicable.

## Nomenclature

SOC	State of charge
V2H	Vehicle-to-home
H2V	Home-to-vehicle
PV	Photovoltaic
EV	Electric vehicle
ESS	Energy storage system
HEMS	Home energy management system
NT	Standard and nominal cell operating temperature conditions
DOD	Total number depth of discharge
*D*	Vehicle travel distance (km)
*i*	Interest rate
*N*	Lifetime
$N_{days}$	Total number of days during the year
γ	Contracted ratio for selling power to the grid
$\lambda_{t}$	Electricity price during different periods of day
$\lambda_{k}$	Peak price
$\lambda_{m}$	Mid-peak price
$\lambda_{off}$	Off-peak price
$\eta_{EV}^{d}$	EV battery discharge efficiency
$\eta_{V}$	Vehicle efficiency (kWh/km)
$\eta_{PV}$	PV generator efficiency
$\eta_{R}$	The PV generator efficiency at reference temperature
$\eta_{ESS}^{c}$	Charging efficiency of ESS
$\eta_{ESS}^{d}$	Discharging efficiency of ESS
$\eta_{EV}^{c}$	Charging efficiency of EV battery
$\eta_{EV}^{d}$	Discharging efficiency of EV battery
β	Binary variable
ρ	Temperature coefficient for cell efficiency
$P_{GL,t}$	Power from the grid-to-load (kW)
$P_{GESS,t}$	Power from the grid-to-ESS (kW)
$P_{GEV,t}$	Power from the grid-to-EV (kW)
$P_{PVG,t}$	Power from the PV-to-grid (kW)
$P_{ESSG,t}$	Power from the ESS-to-grid (kW)
$P_{PVL,t}$	Power from the PV-to-load (kW)
$P_{ESSL,t}$	Power from the ESS-to-load (kW)
$P_{EVL,t}$	Power from the EV-to-load (kW)
$P_{ESS,t}^{c}$	Charging power of ESS (kW)
$P_{ESS,t}^{d}$	Discharging power of ESS (kW)
$P_{EV,t}^{c}$	Charging power of EV (kW)
$P_{EV,t}^{d}$	Discharge power of EV (kW)
$P_{L,t}$	Household power demand at time t
$P_{\max}^{grid}$	Maximum power that can be drawn from the grid (kW)
$P_{\max}^{sell}$	Maximum power that can be sold back to the grid (kW)
$Cost_{PV}^{\mathrm{capital}}$	Daily capital cost of the PV installation
$Cost_{ESS}^{\mathrm{capital}}$	Daily capital cost of ESS installation
$Cost_{EV}^{degradation}$	EV battery degradation cost due to V2H
$C_{PV}$	One-time installation cost of PV (USD/kW)
$C_{ESS}$	One time installation cost of ESS (USD/kWh)
$C_{EV}$	Replacement cost of EV battery
$C_{f}$	Full cycles number during battery lifespan
$Z_{PV}$	Rated capacity of PV array size (kW)
$Z_{ESS}$	Rated capacity of ESS (kWh)
$Z_{EV}$	Rated capacity of EV battery (kWh)
$E_{EV}^{R}$	Required energy for the EV travel distance in the typical day (kWh)
$I_{PV}$	Solar radiation (kWh/m2)
$I_{PV,NT}$	The average hourly solar irradiation incident on the array at NT
$T_{A}$	Ambient temperatures
$T_{R}$	Reference cell temperature
$T_{C}$	The cell temperature
$T_{C,NT}$	Cell temperatures at NT
$T_{A,NT}$	Ambient temperatures at NT
$SOC_{ESS,t}$	ESS SOC at time t
$SOC_{ESS,t-1}$	SOC of ESS in past period
$SOC_{ESS,\max}$	Maximum SOC of ESS
$SOC_{ESS,\min}$	Minimum SOC of ESS
$SOC_{EV,t}$	EV battery SOC at time t
$SOC_{EV,t-1}$	State of charge of EV battery at the past period
$SOC_{EV,\max}$	Maximum SOC of EV battery
$SOC_{EV,\min}$	Minimum SOC of EV battery
$SOC_{EV,R1}$	SOC required for the EV trip distance at the first departure time $t_{d1}$
$SOC_{EV,R2}$	SOC required for the remaining EV trip distance at the second departure time $t_{d2}$
$t_{d1}$	First departure time
$t_{d2}$	Second departure time
$X_{m,best,t}$	Variable m best value obtained for the best candidate
$X_{m,worst,t}$	Variable m worst value obtained for the worst candidate
$X_{m,n,t}^{{}^{\prime }}$	Modified value of $X_{m,n,t}$
$r_{1}$ and $r_{2}$	Are random values between zero and one
